# The efficacy of tucatinib-based therapeutic approaches for HER2-positive breast cancer

**DOI:** 10.1186/s40779-022-00401-3

**Published:** 2022-07-13

**Authors:** Zaid Sirhan, Anita Thyagarajan, Ravi P. Sahu

**Affiliations:** Department of Pharmacology and Toxicology, Boonshoft School of Medicine Wright State University, Dayton, OH 45435 USA

**Keywords:** HER2-positive (HER2+), Breast cancer, Targeted therapy, Tucatinib, Immunotherapy

## Abstract

Overexpression of human epidermal growth factor receptor 2 (HER2) occurs in approximately 15–20% of breast cancer cases. HER2 is a member of the epidermal growth factor receptor (EGFR) family with tyrosinase kinase activity, and its overexpression is linked to poor prognosis and shorter progression-free survival (PFS) and overall survival (OS). Among various treatment options, HER2-targeting monoclonal antibodies and tyrosine kinase inhibitors (TKIs) have mostly been applied in recent decades to treat HER2-positive (HER2+) breast cancer patients. Although positive clinical outcomes were documented in both advanced disease and neoadjuvant settings, the development of resistance mechanisms to such approaches has been one of the major challenges with the continuous usage of these drugs. In addition, patients who experience disease progression after treatment with multiple HER2-targeted therapies often have limited treatment options. The Food and Drug Administration (FDA) has recently approved a new TKI (i.e., tucatinib) for use in combination with immunotherapy and/or chemotherapeutic agents for the treatment of advanced-stage/metastatic HER2+ breast cancer. This review highlights recent updates on the efficacy of tucatinib-based therapeutic approaches in experimental models as well as in the clinical settings of HER2+ breast cancer.

## Background

Breast cancer is one of the most frequently diagnosed human malignancies worldwide and the second most common cause of cancer-related mortality among females in the United States [1–3]. Breast cancer is categorized into four primary clinically relevant molecular subtypes based on the prognosis and levels of gene expression: luminal A or hormone receptor-positive (HR+)/human epidermal growth factor receptor 2-negative (HER2−), luminal B or HR+ /HER2+, HER2+, and triple-negative breast cancer (TNBC) or HR−/HER2− [4–7]. In addition, a fifth subtype that closely resembles luminal A is known as normal-like breast cancer [8]. This review is focused on HER2+ breast cancer. Importantly, the most common modifiable and non-modifiable risk factors associated with breast cancer include lifestyle factors (such as alcohol use and tobacco smoking) and other factors [such as age, body weight, breast density, benign breast conditions, hormonal intake (e.g., oral contraceptives), menopausal status, and family history of malignancy] [9–15].

HER2 has been implicated in mammary carcinogenesis induction in early studies using in vitro and in vivo model systems [16, 17]. HER2 and other members (e.g., HER3/4) of the epidermal growth factor receptor (EGFR) family are receptor tyrosine kinases, which are located on the cellular membrane and respond to a wide variety of ligands [18, 19]. Except for HER2, all other isoforms have a ligand. Activation (i.e., phosphorylation) of HER2 is dependent on homodimerization (i.e., HER2-HER2 homodimer) or heterodimerization with other HER receptors (i.e., dimerization partners), such as HER2-EGFR, HER2-HER3, and HER2-HER4 [20–22]. This activation induces downstream signaling pathways, such as the phosphatidylinositol 3-kinase/protein kinase B (PI3K/AKT) and Ras/Raf/mitogen-activated protein kinase (Ras/MAPK) pathways, which are related to increased cell proliferation and survival as well as augmentation of primary tumor growth and tumor metastasis [19, 23, 24].

Importantly, *HER2* gene is overexpressed or amplified in approximately 15–20% of breast cancer patients, which is linked with poor prognosis, tumor relapse, and worse outcomes, such as shorter progression-free survival (PFS) and overall survival (OS) [20, 25, 26]. Several agents possessing anticancer properties, including taxanes and capecitabine, have been evaluated for the treatment of HER2+ breast cancer [27–30]. However, the efficacy of different therapeutic regimens has been shown to have low-to-moderate response rates and is associated with adverse side effects [20, 24, 31]. One of the possible explanations for these adverse events is that most therapeutic agents target multiple cellular pathways in addition to HER2 signaling, contributing to the development of tumor resistance mechanisms and resulting in reduced effectiveness of such approaches [20, 24, 31]. This has led to the development of targeted therapy with specific targetability against HER2+ breast cancer cells.

Although monoclonal antibodies (e.g., trastuzumab) and tyrosine kinase inhibitors (TKIs) (e.g., lapatinib) have mostly been used in recent decades to treat HER2+ breast cancer patients [32–36], current regimens typically use a combination of taxanes, trastuzumab, and pertuzumab as first-line agents, with ado-trastuzumab emtansine (T-DMI) used as a second-line agent. TKIs (e.g., tucatinib and lapatinib) have also been reserved for use in combination with chemotherapy or HER2-targeted therapy as third-line agents [37–40]. Previous review articles have covered the mechanisms of other HER2-targeting immunotherapies and TKIs [41–45]. Therefore, to avoid repetition, we discuss the mechanisms of one of the most commonly used immunotherapies (e.g., trastuzumab) and recently FDA-approved TKI (i.e., tucatinib) along with their resistance mechanisms, as this combination has been explored in most clinical studies [33, 46, 47]. We also highlight the therapeutic efficacy of tucatinib combination approaches in experimental models and clinical settings of HER2+ breast cancer.

## Mechanisms of action and resistance to trastuzumab and tucatinib

### Mechanisms of action and resistance to trastuzumab

Trastuzumab was one of the first drugs used to target HER2 receptors, which led to a significant improvement in therapeutic efficacy, including increased PFS and OS for HER2+ breast cancer patients. Trastuzumab exerts its effect by binding to the extracellular and juxtamembrane domains of the HER2 receptor. Binding to the extracellular domain blocks HER2 proteolytic cleavage and triggers an immune-mediated response against HER2-overexpressing tumor cells, whereas binding to the juxtamembrane domain selectively blocks HER2-HER3 dimerization independently of a ligand [32, 48–50]. Trastuzumab’s mechanism of action involves antibody-dependent cell cytotoxicity (ADCC), which is dependent on the frequency of CD16 and CD56 co-expressing lymphocytes. Overall, it has been shown that the amount and lytic effectiveness of CD16^+^ lymphocytes are important determinants in trastuzumab-induced ADCC, indicating a potential mechanism for the short-term clinical benefit of trastuzumab as a monotherapy [51]. Another critical factor contributing to the therapeutic efficacy of trastuzumab is the cyclin-dependent kinase inhibitor p27^kip1^ [52]. In this regard, a study performed by Nahta et al. [52] exploited two cellular models known as trastuzumab-resistant (TR) pools generated from SKBR3 HER2-overexpressing breast cancer cells to determine trastuzumab-mediated cytotoxicity escape mechanisms. According to the data, TR cells exhibited an extended S-phase of the cell cycle, resulting in an increased growth rate measured by doubling time compared to the parental cells, which was accompanied by decreased p27^kip1^ expression and increased cyclin-dependent kinase 2 (CDK2) activity. Notably, induced expression of p27^kip1^ was found to enhance the sensitivity of TR cells to trastuzumab, indicating that downregulation of p27^kip1^ might be associated with the development of resistance to trastuzumab therapy.

Despite promising clinical benefits in both advanced disease and neoadjuvant settings [32–36], a substantial proportion of HER2+ breast cancers show a proclivity toward resistance or become refractory to HER2-targeted therapies, including trastuzumab, which has been a major challenge with the use of these drugs [53, 54]. Along similar lines, patients have experienced disease progression after treatment with multiple HER2-targeted therapies, with often limited treatment options [25, 55, 56]. Several mechanisms have been proposed to explain the development of resistance to trastuzumab, such as HER2 signal transduction via activation of the PI3K-AKT-mTOR pathway [57], leading to the accumulation of p95-HER2, which modifies and adapts the signaling from alternative receptors. Another mechanism of resistance involves a mutation in the HER2 structural protein or elevation of other receptor tyrosine kinases, such as insulin-like growth factor receptor [21, 54, 58–61]. Therefore, resistance to HER2 immunotherapy may involve genetic or epigenetic changes that modulate the receptor to be degraded.

**Fig. 1:**
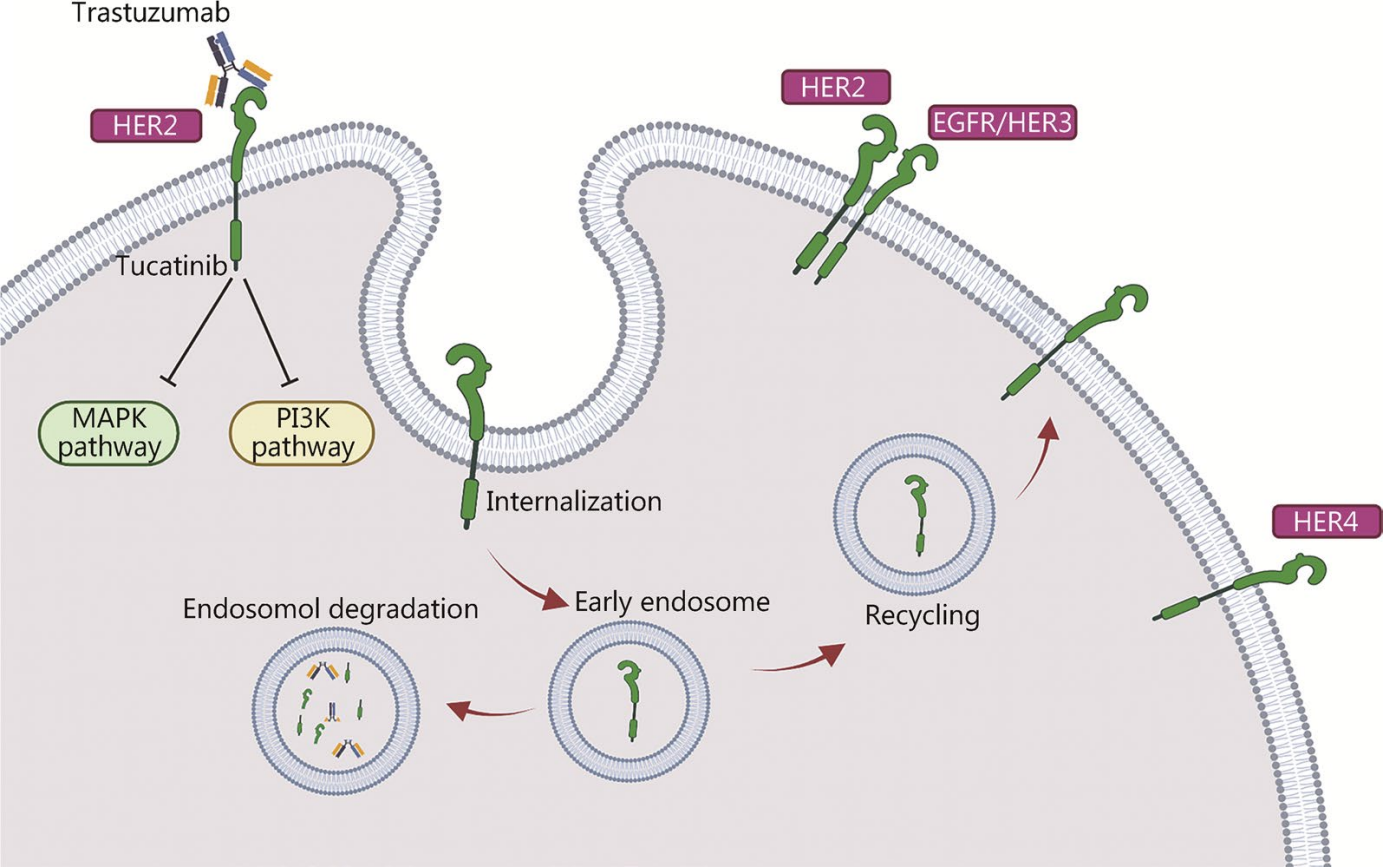
Mechanisms of HER2 internalization and recycling by trastuzumab mediated via different processes resulting in endocytosis and subsequent degradation/recycling. The mechanism of action of tucatinib is mediated via inhibition of HER2 and HER3 phosphorylation, resulting in blockade of the downstream MAPK and AKT signaling pathways and leading to decreased cell proliferation. HER2/3/4 human epidermal growth factor receptor 2/3/4, EGFR epidermal growth factor receptor, MAPK mitogen-activated protein kinase, PI3K phosphatidylinositol 3-kinase

Similarly, HER2 heterodimerization with EGFR/HER3 has been shown to mediate antibody-induced internalization, resulting in subsequent ubiquitination and proteolysis (Fig. 1). Notably, this internalization is the mechanism by which HER2-specific antibodies disable the transforming activity of the receptor [24]. In addition, clathrin-mediated endocytosis of cell surface proteins from the endosomal compartment to the lysosome is responsible for HER2 internalization and degradation [59]. Other trafficking mechanisms also play critical roles in HER2 endocytosis. For example, as the dynamic surface pool of the HER2 receptor is created by varying rates of endocytosis and recycling, trastuzumab efficacy is disrupted when the HER2 surface pool is diminished [62]. It was found that the caveolin-1 (CAV1) protein is important for HER2 cell membrane dynamics during receptor endocytosis. Using in vivo biological models and fresh human tumor cells, Pereira et al. [62] demonstrated that temporal CAV1 reduction with lovastatin enhances the HER2 half-life and its availability at the cell membrane, resulting in better trastuzumab binding to the extracellular HER2 receptor domain.

It has also been demonstrated that sortilin-related receptor 1 (SORLA) interacts with and regulates HER2 subcellular localization by enhancing dynamic recycling of the endosomal receptor back to the cell surface, and levels of SORLA protein and HER2 correlate positively in malignant cells. Importantly, SORLA protein silencing results in decreased HER2 signaling and localization to late endosomes or lysosomes due to impaired normal lysosome function. This mechanism impacts tumor development as well as the resistance/sensitivity of HER2 targeting antibody approaches [63].

Overall, HER2 endocytosis impacts the efficacy of anti-HER2 therapies such as trastuzumab. Study utilizing NSCLC cell lines and patient-derived xenograft models has found ubiquitination and internalization of the HER2 receptor to be the critical mechanisms of endocytosis, augmenting the therapeutic efficacy of anti-HER2 antibody–drug conjugates (ADC), including T-DMI, as well as its synergy with irreversible pan-HER inhibitors [64].

### Mechanisms of action and resistance to tucatinib

Tucatinib is an oral, potent, and selective HER2-specific targeting agent that was approved by the FDA in April 2020 for the treatment of advanced unresectable or metastatic breast cancer. In a preclinical study and phase I clinical trials, tucatinib showed promising activity as a single agent, with improved effectiveness when combined with chemotherapy or trastuzumab [65–69]. Insight into the signaling mechanism revealed that unlike other TKIs, such as lapatinib and neratinib, which act as dual inhibitors for both EGFR and HER2, tucatinib is a specific and reversible inhibitor of the protein tyrosine kinase (PTK) activity of HER2 and exerts minimal inhibition of EGFR [69–71]. Kaur et al. [72] performed an in silico study to determine the molecular basis of synergism between tucatinib and trastuzumab. Given that the mechanism of trastuzumab action occurs via HER2 extracellular domain IV binding, the authors explored the basis of tucatinib action, particularly addressing whether tucatinib binds to the intracellular HER2 tyrosine kinase domain, which occurs intracellularly [73, 74]. Of note, the synergism of combination therapy is achieved when one drug binds to the extracellular domain and the other to the intracellular domain of the HER2 receptor [72]. By using a web-based docking tool, the authors docked tucatinib at the ATP binding site of the HER2 tyrosine kinase domain, and the data showed that tucatinib binds to the tyrosine kinase domain with substantial stability and binding energy. These studies indicate one of the possible reasons for the synergism between trastuzumab and tucatinib resulting in improved clinical responses in HER2+ breast cancer patients [72].

However, another study explored the resistance mechanisms of TKIs, including tucatinib, through gene profiling of different HER2- and non-HER2-overexpressing breast cancer cell lines [75], and identified three novel markers relevant to TKI sensitivities: V-set domain-containing T-cell activation inhibitor 1 (VTCN1), cyclin-dependent kinase 12 (CDK12), and Ras-related C3 botulinum toxin substrate 1 (RAC1). In addition, adenomatous polyposis coli (APC) mutations have been suggested as markers of tucatinib resistance [75]. The efficacy of tucatinib as a single agent vs. in combination with other therapies, such as trastuzumab and capecitabine, of HER2+ breast cancer is summarized below.

## Cellular, preclinical and clinical studies of tucatinib and tucatinib combination approaches

### Evidence from in vitro and in vivo studies

Several studies have evaluated the effects of tucatinib and its combination with other anticancer agents in various experimental models of HER2+ breast cancer. For example, Kulukian et al. [69] demonstrated that tucatinib treatment specifically decreases phosphorylation (i.e., activation) and viability in HER2-expressing BT-474 cells in a dose-dependent manner compared to EGFR-expressing A431 cells. Importantly, tucatinib was found to inhibit not only activation of the HER2 receptor but also binding with its heterodimer partner HER3 receptor, as well as downstream signaling cascades such as MAPK (i.e., MEK and ERK) and PI3K/AKT. Such decreased phosphorylation of AKT, as well as induction of apoptosis, were found to have additive effects in combination with trastuzumab, the HER2-targeted immunotherapy [69]. Preclinical data showed that tucatinib alone suppresses the growth of subcutaneously implanted BT-474 tumor xenografts in a dose-dependent manner compared to vehicle control-treated mice, and enhanced tumor growth suppression was observed with the combination of trastuzumab or docetaxel chemotherapy [69]. Relatively similar in vivo tumor growth-suppressive effects of tucatinib, trastuzumab, and the combination of tucatinib and trastuzumab have also been observed in other HER2+ tumor models, such as gastric, colorectal, and esophageal cancers [69]. Overall, these findings indicate the promising applicability of tucatinib in diverse HER2+ tumor models and that enhanced antitumor efficacy can be achieved via tucatinib in combination with immunotherapy or chemotherapy.

Along similar lines, Conlon et al. [75] compared the antiproliferative efficacy of three TKIs with regard to drug response and gene profiling using different breast cancer cell lines. The authors compared the efficacies and IC_50_ values of neratinib, lapatinib, and tucatinib with other anticancer agents. The tucatinib profile was found to be similar to that of trastuzumab for HER2 selectivity. Additionally, neratinib, lapatinib, and tucatinib were effective against HER2-overexpressing breast cancer models. Neratinib showed the greatest potency; tucatinib exhibited high selectivity for HER2-amplified cell lines but minimal effect on HER2 mutant cell lines [75].

### Evidence from clinical studies

#### Evaluation of tucatinib efficacy with or without other agents in HER2+ breast cancer patients previously treated with HER2-targeted therapies

Multiple studies have explored the efficacy of tucatinib in combination with chemotherapeutic agents or immunotherapy in HER2+ breast cancer patients who were previously treated with HER2-targeted therapies. A summary of these studies is presented in Table 1.

**Table 1:** Summary of clinical studies evaluating tucatinib or its combination with other agents in HER2+ metastatic breast cancer patients

Treatment(s)	Other factor(s)	Study design and finding(s)
Tucatinib [65]	–	To determine the MTD, antitumor activity, and pharmacokinetic properties of tucatinib The tucatinib MTD was determined to be 600 mg twice daily; 22% of patients treated at the MTD had a partial response + SD
Tucatinib + trastuzumab + capecitabine [33]	ALT/AST	To evaluate the therapeutic responses of tucatinib vs. placebo combination with trastuzumab and capecitabine Tucatinib-combination resulted in improved PFS and increased OS with common adverse events compared to the placebo-combination group

*HER2*+ HER2-positive, *MTD* maximum tolerable dose, *SD* stable disease, *ALT* alanine aminotransferase, *AST* aspartate aminotransferase, *PFS* progression-free survival, *OS* overall survival

In a phase I dose-escalation study, Moulder et al. [65] evaluated the maximum tolerable dose (MTD), antitumor activity, and pharmacokinetic properties of tucatinib. The MTD was found to be 600 mg twice daily, with dose-limiting toxicity at 800 mg twice daily. In addition, tucatinib had a favorable pharmacokinetic profile. When given at the MTD, tucatinib maintained the mean steady-state concentration at or above the predicted IC_90_ value for HER2 inhibition [65]. The overall median T_max_ for tucatinib was approximately 2 h, and the overall median half-life was more than 5 h. No significant food effect was observed for tucatinib or metabolite exposure; however, variability among patients was high [65]. Of all patients treated with MTD doses, 22% had a partial response with stable disease (SD) when evaluated at ≥ 24 weeks, but 28% experienced disease progression [65].

In the HER2CLIMB study, Murthy et al. [33] determined the therapeutic responses of the tucatinib combination in patients who received prior treatments involving trastuzumab, pertuzumab, and trastuzumab emtansine and experienced disease progression. The patients were assigned randomly into two groups: tucatinib-combination group (administered tucatinib in combination with trastuzumab and capecitabine) and placebo-combination group (treated with a placebo in combination with trastuzumab and capecitabine). The primary endpoint was PFS at the one-year time point, which was found to be 33.1% in the tucatinib-combination group and 12.3% in the placebo-combination group, with a median PFS of 7.8 months and 5.6 months, respectively [33]. OS after two years was 44.9%, with a median OS of 21.9 months in the tucatinib-combination therapy group compared to 26.6% and 17.4 months in the placebo-combination group. Notably, patients in the tucatinib-combination group experienced common adverse effects of grade 3 or higher, including diarrhea, fatigue, and increased levels of alanine aminotransferase (ALT) and aspartate aminotransferase (AST) compared to the placebo-combination group. Overall, the findings indicate that tucatinib combined with trastuzumab and capecitabine can be used to decrease the risk of disease progression or to increase survival benefits in HER2+ metastatic breast cancer patients with or without brain metastasis [33].

#### Evaluation of tucatinib combination in HER2+ breast cancer patients with brain metastasis

The incidence rate of breast cancer with brain metastasis ranges from 10 to 30% and is associated with significant morbidity and mortality [70, 76]. Therefore, several studies have evaluated the efficacy of tucatinib combination therapy in these patients. A summary of these studies is presented in Table 2.

**Table 2:** Summary of clinical studies evaluating tucatinib combination approaches in HER2+ metastatic breast cancer patients with brain metastasis

Treatment(s)	Parameter(s)	Study design and finding(s)
Tucatinib + trastuzumab + capecitabine [47]	OS	To evaluate safety. The OS was 20.7 months with the tucatinib-combination
Tucatinib + T-DMI [77]	mPFS	Phase I dose-escalation study to evaluate effect. The mPFS was 8.2 months
Tucatinib + trastuzumab [78]	MTD	Phase I dose-escalation study to evaluate response. The combination therapy was tolerable and effective in HER2+ breast cancer patients having brain metastases
Tucatinib + capecitabine, tucatinib + trastuzumab, tucatinib + capecitabine + trastuzumab [46]	mPFS	Phase I dose-escalation study to evaluate response. ORR of tucatinib + capecitabine was 83%, tucatinib + trastuzumab was 40%, and tucatinib + capecitabine + trastuzumab was 61%. The mPFS of the triple combination in patients with no brain metastasis was 7.8 months; however, it was 6.7 months in patients with brain metastasis
Tucatinib + trastuzumab + capecitabine [79]	OS	Phase II study to evaluate safety and efficacy. The median OS was 11.9 months and the median time to CNS progression 6.9 months

*HER2*+ HER2-positive, *OS* overall survival, *T-DMI* ado-trastuzumab emtansine, *mPFS* median progression-free survival, *MTD* maximum tolerable dose, *ORR* objective response rate, *SD* stable disease, *CNS* central nervous system

In a subset of the HER2CLIMB study, Lin et al. [47] evaluated the effects of tucatinib in combination with trastuzumab and capecitabine in breast cancer patients who were previously treated or remained untreated with progressing brain metastasis. In patients not previously treated, OS in the tucatinib combination group was 20.7 months compared with 11.6 months in the placebo combination group. It was also found that the tucatinib-combination regimen delayed the time between the initial and second brain progression by 7.6 months compared with 3.1 months in the placebo combination group [47].

Borges et al. [77] conducted an open-label phase Ib clinical trial with the primary objective of evaluating the MTD of tucatinib in combination with T-DMI in T-DMI-naive advanced-stage metastatic HER2+ breast cancer patients, including those who had brain metastasis. The secondary objectives of this combination were determination of the safety, tolerability, pharmacokinetics, and preliminary antitumor activity, as assessed by the objective response rate (ORR) and PFS. The MTD of tucatinib was 300 mg twice daily, with dose-related toxic effects observed at 350 mg when given twice a day. Overall, the ORR of tucatinib combined with T-DMI at the MTD was 48%, and the median PFS (mPFS) was 8.2 months; the mPFS of patients previously treated with trastuzumab and pertuzumab was 6.5 months [77]. The clinical benefit rate of tucatinib combination therapy in patients, which was defined by the complete response, partial response, or stable response for over 6 months, was 58%, with a median response duration of 6.9 months. Among patients with brain metastasis, the mPFS of tucatinib combination therapy was 6.7 months, with a median overall response duration of 6.9 months, as per the response evaluation criteria in solid tumors (RECIST) version 1.1. Notably, the three most common adverse events observed were nausea, diarrhea, and fatigue [77].

Along similar lines, in a phase I study, Metzger Filho et al. [78] evaluated response to tucatinib when combined with trastuzumab. The primary objective of the study was to determine the MTD; the secondary objective included responses such as intracranial and extracranial parameters using modified RECIST and the clinical benefit rate. The study evaluated tucatinib MTD of 750 mg given once daily in cohort B, and a dose-limiting grade 3 adverse effect (i.e., transaminitis) was observed in one patient. Importantly, the combination regimen was found to be tolerable and effective in HER2+ breast cancer patients having brain metastases. The most common dose-limiting toxicities included thrombocytopenia and elevation of ALT/AST [78].

The MTD, safety, tolerability, and antitumor activity of tucatinib in combination with capecitabine and trastuzumab were evaluated in another phase I open-label nonrandomized dose-escalation study [46]. For this study, the authors recruited HER2+ breast cancer patients with or without brain metastasis who were previously treated with trastuzumab, pertuzumab, or trastuzumab emtansine. The tucatinib MTD was determined to be 300 mg twice daily. The ORR of the tucatinib and capecitabine combination was 83%, that of the tucatinib and trastuzumab combination was 40%, and that of the tucatinib, capecitabine, and trastuzumab combination was 61% [46]. Importantly, the mPFS for patients treated with the triple combination was 7.8 months compared to 7.1 months with tucatinib and capecitabine and 5.5 months with tucatinib and trastuzumab. Moreover, the median response duration was 5.2 months with tucatinib and capecitabine compared to 8.9 months with tucatinib and trastuzumab and 11.0 months with tucatinib, capecitabine, and trastuzumab [46]. Notably, 56% of patients enrolled at the time of the study had brain metastasis. Of these, 59% were either untreated for brain metastasis or had previously been treated, with progressive brain metastasis. The mPFS of the patients with brain metastasis treated with the combination of tucatinib, capecitabine, and trastuzumab was 6.7 months. Adverse effects reported irrespective of dose, causality, and grade were fatigue, diarrhea, and palmar-plantar erythrodysesthesia syndrome [46].

The same combination regimen was also evaluated for its safety and efficacy in HER2+ breast cancer patients with leptomeningeal metastasis (LM) who had poor prognosis and limited therapeutic options. In this nonrandomized phase II clinical trial, Murthy et al. [79] enrolled 17 adult HER2+ breast cancer patients based on Karnofsky performance status (KPS) of > 50 who were newly diagnosed with untreated LM, as confirmed by positive cerebrospinal fluid (CSF) cytology and/or radiographic evidence of LM plus other signs and symptoms. The primary endpoint of this study was OS. The median age of the patients was 53 years old. All patients had radiographic evidence of LM in the brain and were treated for the brain metastases; 8 patients had abnormal CSF cytology. The results showed a median OS of 11.9 months and a median time to central nervous system (CNS) progression of 6.9 months. These findings for the first time indicated the clinical benefits of tucatinib, trastuzumab, and capecitabine for the treatment of HER2+ metastatic breast cancer patients with LM. Importantly, a recent highlight published by the American Society of Clinical Oncology (ASCO) suggested that the combination of tucatinib with trastuzumab and capecitabine constitutes a novel standard therapy for pretreated HER2+ breast cancer patients with active brain metastasis [80].

In addition to HER2+ metastatic breast cancer, this combination regimen has been evaluated in other human HER2+ malignancies, including metastatic colorectal cancer (mCRC). For example, the MOUNTAINEER open-label phase II study determined the safety and efficacy of tucatinib, trastuzumab, and capecitabine combination in HER2+ *RAS* wild-type mCRC, and interim analysis of 26 patients showed an ORR of 52.2%, mPFS of 8.1 months, and mOS of 18.7 months [81]. Of significance, the MOUNTAINEER-02 phase II/III study is ongoing (NCT04499924) and aims to evaluate the safety and efficacy of tucatinib and trastuzumab combined with ramucirumab and paclitaxel in patients with gastric or gastroesophageal junction adenocarcinoma (GEC) [82].

## Conclusions

Several studies have suggested that the combination of tucatinib with trastuzumab and capecitabine exhibits increased effectiveness over trastuzumab and capecitabine alone in treating HER2+ breast cancer patients. However, despite an increased therapeutic efficacy of combination therapies, one of the ongoing challenges is therapy-induced adverse effects, indicating the mechanisms involved in mediating such effects, which should be investigated. As tucatinib is a relatively new FDA-approved targeted therapy, its efficacy with other combinations should also be explored to assess their overall effectiveness and compare it with published data. Importantly, some clinical trials are ongoing and recruiting patients to evaluate tucatinib in combination with other anticancer agents.

## Data Availability

Not applicable.

## Abbreviations

ADCC: Antibody-dependent cell cytotoxicity
ADC: Antibody–drug conjugates
ALT: Alanine aminotransferase
APC: Adenomatous polyposis coli
ASCO: American Society of Clinical Oncology
AST: Aspartate aminotransferase
CAV1: Caveolin-1
CDK2: Cyclin-dependent kinase 2
CDK12: Cyclin-dependent kinase 12
CNS: Central nervous system
CSF: Cerebrospinal fluid
EGFR: Epidermal growth factor receptor
FDA: Food and Drug Administration
GEC: Gastroesophageal junction adenocarcinoma
HER: Human epidermal growth factor
HER2+: HER2-positive
KPS: Karnofsky performance status
LM: Leptomeningeal metastasis
mCRC: Metastatic colorectal cancer
mPFS: Median progression-free survival
MTD: Maximum tolerable dose
ORR: Objective response rate
OS: Overall survival
PFS: Progression-free survival
PI3k/AKT: Phosphatidylinositol 3-kinase/protein kinase B
PTK: Protein tyrosine kinase
RAC1: Ras-related C3 botulinum toxin substrate 1
Ras/MAPK: Ras/Raf/mitogen-activated protein kinase
RECIST: Response evaluation criteria in solid tumors
SD: Stable disease
SORLA: Sortilin-related receptor 1
T-DMI: Ado-trastuzumab emtansine
TKI: Tyrosine kinase inhibitor
TNBC: Triple-negative breast cancer
TR: Trastuzumab-resistant
VTCN1: V-set domain-containing T-cell activation inhibitor 1
